# Esquadrinhando corpos, mutilando histórias: as psicocirurgias aplicadas nas mulheres internadas no Hospício de Juquery

**DOI:** 10.1590/S0104-59702024000100036

**Published:** 2024-07-26

**Authors:** Gustavo Querodia Tarelow

**Affiliations:** i Pesquisador, Museu Histórico/Faculdade de Medicina/Universidade de São Paulo. São Paulo – SP – Brasil gustarelow@gmail.com

**Figure:**
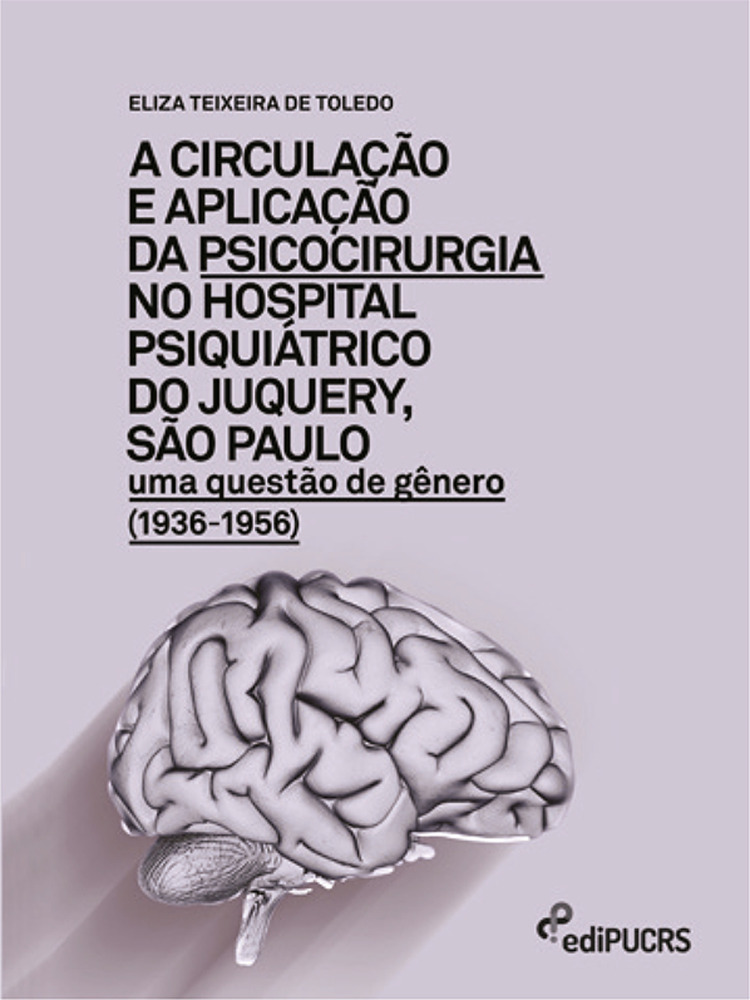
TOLEDO, Eliza Teixeira de. A circulação e aplicação da psicocirurgia no Hospital Psiquiátrico do Juquery, São Paulo: uma questão de gênero (1936-1956). Porto Alegre: EdiPUCRS, 2022. 396p.

No último quartel do século XIX, especialmente após a instauração do regime republicano e a consequente ascensão das elites cafeeiras ao centro do poder, São Paulo se tornou um estado com dinâmica social, política e econômica cada vez mais complexa. Buscando equacionar as contradições do processo de rápida expansão urbana e populacional, a administração paulista estabeleceu uma estrutura de serviços e instituições que visava garantir a produtividade da massa trabalhadora e uma ordem social esculpida segundo o *status quo* vigente. Nesse contexto, sob a direção de Franco da Rocha e o endosso dos membros da Sociedade de Medicina e Cirurgia de São Paulo, foi inaugurado, em 1898, o Hospício de Juquery, erigido nos moldes do alienismo francês e com a pretensão de ser uma instituição modelar para o país.

Verdadeiro “espelho do mundo”, como bem definiu Maria Clementina Cunha (1986), o Juquery buscou, ao menos em seus primeiros 50 anos de funcionamento, ombrear com os principais centros internacionais de estudos sobre a psiquiatria, ao passo que foi a ponta de lança de uma política de contenção social, segregação e discriminação. Contraditória, profunda e complexa, a história dessa instituição vem sendo perscrutada por pesquisadores sob diversas propostas teórico-metodológicas nas últimas décadas. Oportunamente, essa história acaba de receber delineamentos novos e importantes no livro de Eliza Teixeira de Toledo *A circulação e aplicação da psicocirurgia no Hospital Psiquiátrico do Juquery, São Paulo: uma questão de gênero (1936-1956)*.

A obra lança luz sobre o processo de desenvolvimento e aplicação de diversas técnicas de mutilação cerebral empreendidas com fins terapêuticos, chamadas de “psicocirurgias”, que, segundo a autora, podem ser designadas como “um conjunto de métodos cirúrgicos em um cérebro anatomicamente normal para o tratamento de doenças mentais e comportamentos inadequados” (Toledo, 2022, p.20). Tais técnicas, dentre as quais destacam-se a leucotomia, a lobotomia e suas variantes, ganharam reconhecimento internacional, incluindo a atribuição do prêmio Nobel de Medicina a seu idealizador, o afamado psiquiatra português Egas Moniz. As psicocirurgias foram inseridas no arsenal terapêutico do Juquery em 1936, em um contexto de profusão de terapias de choque, como malarioterapia, insulinoterapia, convulsoterapia, bem como de uma compreensão sobre os transtornos mentais pautada pelo organicismo e pela eugenia (Tarelow, 2013, p.16).

Eliza Toledo apresenta, no entanto, um passo adiante na compreensão da aplicação das psicocirurgias, ao demonstrar as especificidades da dimensão de gênero presentes no uso em larga escala dessas técnicas nos indivíduos internados no Juquery ao longo dos 20 anos analisados em seu trabalho. De acordo com a autora, na ala denominada “Hospital Central”, lócus da aplicação terapêutica no Juquery (em oposição às “Colônias”, para onde os pacientes considerados “cronificados” eram enviados), 62% dos pacientes eram do sexo feminino. No entanto, ao observar a totalidade das psicocirurgias aplicadas no período e registradas nos prontuários médicos analisados, constatou que 92% dos indivíduos submetidos a tais técnicas eram mulheres. Esses dados, segundo a autora, “evidenciam que, ainda que o Hospital Central fosse um espaço onde prevalecesse o investimento em processos terapêuticos, em relação à psicocirurgia houve uma clara predominância das operações em pacientes do sexo feminino” (Toledo, 2022, p.241).

Com isso em vista, o livro, ao longo de seus quatro capítulos, faz um percurso sobre a conformação da psiquiatria organicista, sobre o contexto histórico da sociedade paulista do segundo quartel do século XX, dedicando especial atenção às questões endógenas do Juquery no que tange ao perfil de seus psiquiatras e pacientes. Desse modo, Eliza Toledo lança luz sobre personagens destacados nesse contexto que, até então, careciam de maior atenção da historiografia, como, a título de exemplo, os médicos Mário Yahn e Aloysio de Mattos Pimenta. Ambos desenvolveram variações das técnicas propostas por Egas Moniz e angariaram prestígio entre seus pares ao publicar, em importantes periódicos nacionais e internacionais, uma série de artigos sobre as psicocirurgias, aplicadas invariavelmente em mulheres.

A autora empreende suas análises a partir de uma abordagem teórico-metodológica operada de forma complementar e dialógica, tendo como eixo central os estudos de gênero e feministas. Além disso, faz uso das teorias ator-rede de Bruno Latour e dialoga com os conceitos de “racionalidade terapêutica”, de Joel Braslow, e de “corpo experimental”, de Ilana Löwy. Quanto às fontes, Toledo apresenta profunda pesquisa documental nos prontuários médicos dos indivíduos internados no Juquery entre 1936 e 1956, além de manejar uma série de artigos científicos publicados pelos psiquiatras paulistas no período citado. Com esse conjunto de fontes e referenciais, a autora busca responder, entre outras, às seguintes questões:

Enquanto técnica experimental, a psicocirurgia foi ali utilizada em que perfil de pacientes, em função de que patologias e/ou sintomas? Ao fim, existiu, de fato, um maior número de mulheres operadas naquele hospital? Se sim, que fatores influenciaram essa discrepância numérica? Que correlação o uso da psicocirurgia no Juquery manteve com noções circulantes de gênero? Afinal, o que significava ter uma conduta suficientemente desviada a ponto de a psicocirurgia ser indicada? (Toledo, 2022, p.27).

Finalmente, é possível afirmar que o livro traz contribuição de grande relevância para a historiografia da psiquiatria brasileira. Mais que isso, é leitura fundamental para todos os interessados em compreender como os corpos femininos e os seus comportamentos considerados “desviantes” foram alvo de esquadrinhamento e de intervenções, em muitos casos, irreversíveis, como as cirurgias de mutilação cerebral, visando adequá-las a uma determinada norma estabelecida baseada em expectativas de papéis de gênero erigidas, também sob pressupostos científicos, por uma sociedade patriarcal e machista.
